# Occupational exposure to organic dust and risk of lymphoma subtypes in the EPILYMPH case–control study

**DOI:** 10.5271/sjweh.3925

**Published:** 2020-12-16

**Authors:** Pierluigi Cocco, Giannina Satta, Federico Meloni, Ilaria Pilia, Fahad Ahmed, Nikolaus Becker, Delphine Casabonne, Silvia de Sanjosé, Lenka Foretova, Marc Maynadié, Alexandra Nieters, Anthony Staines, Andrea ‘t Mannetje, Mariagrazia Zucca, Maria Grazia Ennas, Marcello Campagna, Sara De Matteis, Yolanda Benavente

**Affiliations:** 1 Department of Medical Sciences and Public Health, University of Cagliari, Cagliari, Italy; 2 Institute of Public Health, Hacettepe University, Ankara, Turkey; 3 German Cancer Research Center, Heidelberg, Germany; 4 Catalan Institute of Oncology, IDIBELL, Barcelona, Spain; 5 Consorcio Centro de Investigación Biomédica en Red de Epidemiología y Salud Pública, Madrid, Spain; 6 Department of Cancer Epidemiology and Genetics, Masaryk Memorial Cancer Institute and MF MU, Brno, Czech Republic; 7 Dijon University Hospital, Dijon, France; 8 Medical Center, University of Freiburg, Freiburg, Germany; 9 Dublin City University, Dublin, Ireland; 10 Massey University Center for Public Health, Wellington, New Zealand; 11 Department of Biomedical Sciences, University of Cagliari, Cagliari, Italy

**Keywords:** B cell lymphoma, epidemiology, flour dust, Hodgkin lymphoma, leather dust, textile dust, wood dust

## Abstract

**Objectives:**

This study aimed to estimate the risk of lymphoma and its major subtypes in relation to occupational exposure to specific organic dusts.

**Methods:**

We explored the association in 1853 cases and 1997 controls who participated in the EpiLymph case–control study, conducted in six European countries in 1998–2004. Based on expert assessment of lifetime occupational exposures, we calculated the risk of the major lymphoma subtypes associated with exposure to six specific organic dusts, namely, flour, hardwood, softwood, natural textile, synthetic textile, and leather, and two generic (any types) groups: wood and textile dusts. Risk was predicted with unconditional regression modeling, adjusted by age, gender, study center, and education.

**Results:**

We observed a 2.1-fold increase in risk of follicular lymphoma associated with ever exposure to leather dust [95% confidence interval (CI) 1.01–4.20]. After excluding subjects who ever worked in a farm or had ever been exposed to solvents, risk of B-cell lymphoma was elevated in relation to ever exposure to leather dust [odd ratio (OR) 2.2, 95% CI 1.00–4.78], but it was not supported by increasing trends with the exposure metrics. Risk of Hodgkin lymphoma was elevated (OR 2.0, 95% CI 0.95–4.30) for exposure to textile dust, with consistent upward trends by cumulative exposure and three independent exposure metrics combined (P=0.023, and P=0.0068, respectively).

**Conclusions:**

Future, larger studies might provide further insights into the nature of the association we observed between exposure to textile dust and risk of Hodgkin lymphoma.

Lymphoma includes a heterogeneous group of hematological malignancies. The overall combination of lymphoma subtypes ranks fifth of the most common cancer in the developed regions of the world (1). Several epidemiological studies have provided evidence of a link with occupational exposures, such as solvents and pesticides (2, 3), but results on the association with exposure to organic dust are inconsistent (4–10).

Workplace exposure to organic dust is a well-established cause of respiratory disorders. This includes allergic conditions, such as asthma, hypersensitivity pneumonitis, and organic dust toxic syndrome (11), which have shown a moderate inverse association with risk of malignant lymphomas in several case–control studies (12–14). However, a large registry-based cohort study in Sweden reported an increase in risk of non- Hodgkin lymphoma (NHL) and Hodgkin lymphoma (HL) in prick-test positive patients affected by a range of atopy-related diseases (15); also, incident cases of lymphoma did not exceed the expectation in another cohort of patients who tested positive for allergy (16). On the other hand, occupational exposure to seven high molecular weight sensitizing agents, some of which related to organic dust, identified with the aid of a job-exposure matrix (17), was inversely associated with NHL risk (18, 19). Although the mechanism remains elusive, the well-known role of immunological factors in lymphoma development lends credibility to the hypothesis that chronic stimulation of the immune system by endogenous and exogenous exposures, including high molecular weight allergens related to some organic dusts, might protect against risk of developing NHL (18).

In this paper, we investigated the association between exposure to specific organic dusts, selected at the early stage of planning the study based on an extensive literature search on occupational risk factors, namely wood dust (any), hardwood, softwood, textile dust (any), natural textile, synthetic textile, flour dust, and leather dust, and risk of lymphoma overall, B-cell lymphoma, and five major lymphoma subtypes.

## Methods

### Study design and participants

The EPILYMPH study is a multicenter case–control study on the etiology of lymphoma, which was conducted in six European countries, namely the Czech Republic, France, Germany, Ireland, Italy, and Spain, in 1998–2004. A detailed description of the study can be found elsewhere (20). Briefly, eligible cases were consecutive adult patients first diagnosed with any lymphoma subtype during the study period and resident in the referral area of the participating centers. The diagnosis was classified according to the 2001 WHO classification of lymphoma (21), and an international team of pathologists, coordinated by Marc Maynadié, reviewed the slides of about 20% of cases from each center. Controls from Germany and Italy were randomly selected by sampling from registers of the resident population. In the other countries, hospital controls were recruited, with eligibility criteria restricted to diagnoses other than cancer, infectious diseases and immunodeficiency. Both population and hospital controls were frequency matched to the cases by gender, 5-year age group and residence area. Approval from the relevant ethics committees was obtained in all centers. A signed informed consent was obtained directly from the 2348 lymphoma cases and 2462 controls who participated in the study prior to interview and blood withdrawal. Overall, the participation rate was 88% in cases, 81% in hospital controls, and 52% in population controls.

### Questionnaire and occupational history

Trained interviewers gathered information directly from all the cases and controls, using the same standardized questionnaire translated into the language of each country, on socio-demographic factors, lifestyle habits, and lifetime work history. Study subjects who reported having worked in jobs of prior interest, including bakers, woodworkers, and workers in the leather and the textile industry, were administered a job-specific module inquiring in more detail into the exposures of interest. Questions in the job-specific modules referred to work procedures, tasks accomplished, tools used, as well as chemicals, such as glues, paints, dyes, wood preservatives, insecticides, or fungicides, indoor/outdoor work, presence and functioning of ventilation systems if indoor, and use of personal protective equipment.

### Occupational exposure assessment

In each participating center, industrial hygienists, blinded to the case/control status of study subjects, reviewed the questionnaire information for each job in their work history to assess exposure to 43 agents, of prior interest for study, including any wood dust, hardwood dust, softwood dust, any textile dust, natural textile dust, synthetic textile dust, flour dust, and leather dust, using the following exposure metrics:

*- confidence* about the exposure assessment, representing the industrial hygienist’s degree of certainty that the worker had been truly exposed to the agent, based upon two criteria: (i) the probability of the agent occurring while performing the tasks implying exposure (unexposed; exposure possible, but not probable; probable; certain); and (ii) the proportion of workers exposed among those in the same job (1=<40%; 2=40–90%; 3=>90%);
*- intensity of exposure*, expressed on a four-point scale (unexposed; low; medium; high). Agent-specific cut-off points of intensity categories were set based on the respective most recent threshold limit value, or benchmark occupations when no threshold limit value existed. When grouping individual agents, such as any wood dust, or any textile dust, the group intensity level was that of the contributing agent attaining the highest intensity level;
*- frequency of exposure*, representing the proportion of working time involving contact with the agent (unexposed; 1–5% ; 5–30%; >30% work time).

A cumulative exposure score was calculated for each agent as follows (22):

$C_{\mathrm{ij}}=\sum {\left(y_{\mathrm{ij}}*f_{\mathrm{ij}}/3\right)}^{x_{\mathrm{ij}}}$

where *C_i_* is the cumulative exposure score; *i* is the study subject; *j* is the *j^th^* job in the work history of the *i^th^* study subject; y_ij_ is the duration of exposure (in years) of the *j^th^* job of the *i^th^* study subject; x_ij_ is the exposure intensity level the *j^th^* job of the *i^th^* study subject; and f_ij_ is the exposure frequency level in the *j^th^* job of the *i^th^* study subject.

For the purposes of the analysis, cumulative exposure scores were then categorized into quartiles among the exposed cases and controls, corresponding to increasing level of lifetime cumulative exposure. Due to sparse data within each exposure category, in the analysis of lymphoma subtypes, the score of cumulative exposure was categorized as either below or above the median value.

### Statistical methods

We used unconditional logistic regression models to assess risk of lymphoma (all subtypes), B-cell lymphoma, and its major subtypes, including diffuse large B cell lymphoma (DLBCL), chronic lymphocytic leukemia (CLL), follicular lymphoma (FL), and multiple myeloma (MM), as well as HL associated with exposure to each of the specific organic dusts of prior interest. The following covariates were included in the regression models: age (continuous), gender, study center, education (categorized as ≤8, 9–12, ≥13 years), ever worked in a farm, ever exposed to solvents, and ever suffering from atopy, including any previous diagnosis of asthma, eczema, hay fever, or other allergies excluding drug allergies. We also ran regression models with ever occupational exposure to pesticides as a covariate instead of farm work. Consistently with previous papers, we included age, gender, the two variables used for frequency matching controls to cases, and study center as covariates in the regression models to account for the varying proportion of refusals in the participating centers (22), and the varying distribution by age and gender of the specific lymphoma subtypes. In the analyses for this study, we considered farm work, and solvents as possible confounders, being occupational risk factors for several lymphoma subtypes (22, 23), and possibly associated with exposure to one or more of the six organic dusts object of this report. Occupational experts supported by agronomists assessed exposure to pesticides in the same dataset. We extracted the information from previous work (24), and used it as a covariate instead of farm work, to better characterize agricultural confounders.

To maximize the potential for detecting what are presumably weak associations, we excluded all subjects who had ever been exposed to any organic dust from the unexposed cases and controls, so as to have the same unexposed reference when investigating each specific organic dust. After excluding 871 subjects exposed to unspecified organic dust and 89 subjects who did not have any job entry in the work history, 1853 cases and 1997 controls remained available for inclusion in the analysis. We therefore retained as exposed 1112 subjects (551 cases and 561 controls) who had ever been exposed to one or more of the specific organic dusts we considered, and 2738 subjects (1302 cases and 1436 controls) who had never been exposed to the specific organic dusts of interest or any unspecified organic dust. We conducted further analyses by excluding study subjects who had ever been exposed to solvents and subjects who ever worked in a farm or had ever been exposed to pesticides, alternatively, and by type of controls, whether hospital controls or population controls.

We calculated the Wald test for trend with four exposure metrics – intensity, frequency, duration of exposure, and cumulative exposure – after linear transformation of all the covariates in the regression model, and we set the two-sided statistical threshold to reject the null hypothesis at P<0.05. In the analysis, we did not use the confidence metric, as the large majority of study subjects were in the high confidence category (table 1), and the proportion of those classified with medium or high confidence ranged 95.1–100% across the specific organic dusts. The reciprocal independence between the categorical metrics of frequency, intensity and duration of exposure was previously assessed (25), which provided the conditions to apply the Fisher method for combined probability testing to calculate the chance probability associated with a positive trend observed with these three independent tests bearing upon the same overall hypothesis (26). We used the Cochran’s Q test to detect heterogeneity in risk across study centers and across subtypes (26). All the analyses were conducted using SPSS® version 16.0 (IBM, Armonk, NY, USA).

**Table 1:** Distribution of the study population by gender, age, education level, atopy, concurrent occupational exposures and ever exposure to six organic dusts. P-values in the rightmost column are from Chi^2^ tests for the differences by case–control status. [SD=standard deviation.]

Characteristics	Cases ( N=1853)				Controls (N=1997)				P-value
	
	N	%	Mean	SD	N	%	Mean	SD	
Age			56.1	16.18			56.2	16.2	
Gender									
Male	1061	57.2			1110	55.6			0.295
Female	792	42.8			887	44.4		
Education (years)									
≤8	754	40.7			797	39.9			0.849
9–13	801	43.2			881	44.1			
≥13	298	16.1			319	16.0			
Centre									
Spain	291	15.7			351	17.6			
France	214	11.5			195	9.8			
Germany	636	34.3			640	32.0			n.a.
Italy	260	14.0			335	16.8			
Ireland	201	10.9			199	10.0			
Czech Republic	251	13.6			277	13.9			
Atopy (ever diagnosed)	693	37.4			751	37.6			0.894
Concurrent occupational exposures									
Farm work (ever)	260	14.0			288	14.4			0.729
Pesticides (ever exposed)	147	7.9			145	7.3			0.431
Solvents (ever exposed)	785	42.4			822	41.2			0.450
Ever exposed to organic dusts (high confidence) a									
Wood dust (any)	266 (185)	14.4 (69.5)			276 (188)	9.8 (68.1)			0.516
Hardwood	91 (86)	4.9 (94.5)			96 (88)	4.7 (91.7)			0.769
Softwood	155 (125)	8.4 (80.6)			144 (123)	7.1 (85.4)			0.159
Textile dust (any)	216 (184)	11.7 (85.2)			203 (177)	10.0 (87.2)			0.127
Natural textile	176 (155)	9.5 (88.1)			176 (158)	8.6 (89.8)			0.387
Synthetic textile	130 (103)	7.0 (79.2)			127 (85)	6.2 (66.9)			0.352
Flour	66 (55)	3.6 (83.3)			87 (75)	3.6 (86.2)			0.287
Leather	57 (47)	3.1 (82.5)			57 (43)	2.5 (75.4)			0.608
Ever exposed to any of the above organic dusts	551	29.7			561	28.1			0.261

a The number and percentage in brackets refer to the number of subjects classified as exposed with high confidence, and to the percentage of these subjects over the ever exposed, respectively.

## Results

Table 1 shows the distribution of selected variables in the study population. There were no substantial differences by case–control status in the core variables. Also, the proportion of ever exposed to the organic dusts did not vary by case–control status. Among the exposed, subjects in the top level of confidence of exposure were the large majority, ranging 67–94% by type of dust and case–control status. When considering only subjects with high confidence of exposure, being ever exposed to synthetic textile dust, were more prevalent among the cases (P=0.055). There was no difference between cases and controls by having ever been working in a farm, or exposed to pesticides or solvents, known risk factors for lymphoma (P=0.729, P=0.431, P=0.450, respectively). History of atopy did not vary by case–control status (table 1). There was no substantial overlap between exposure to organic dust and exposure to the other known occupational risk factors for lymphoma, namely solvents, and pesticides (data not shown).

Risk of DLBCL was elevated in relation to ever exposure to textile dust [odds ratio (OR) 1.4, 95% confidence interval (CI) 1.00–1.98], and risk of FL was elevated in relation to ever exposure to leather dust (OR=2.1, 95% CI 1.01–4.20) (table 2). However, we did not detect an upward trend in risk of either subtype by increasing cumulative exposure level (P=0.097 and P=0.267, respectively) (table 2).

**Table 2:** Risk for the major lymphoma subtypes associated with ever exposure and P-value associated with the Wald test for trend by cumulative exposure to specific organic dusts. Regression models are adjusted for age (continuous), gender, study centre, education (categorized as ≤8, 9–12, ≥13 years), ever worked in a farm, ever exposed to solvents, and ever suffering from atopy. [OR=odds ratio; CI=confidence interval.]

Exposure	Cases/controls	OR	95% CI	P-value for trend
All lymphomas				
Unexposed	1302/1436	1.0	-	-
Wood dust (any)	266/276	1.0	0.80–1.22	0.387
Hardwood dust	91/96	1.0	0.70–1.31	0.958
Softwood dust	155/144	1.1	0.82–1.36	0.359
Textile dust (any)	216/203	1.2	0.94–1.46	0.194
Natural textile dust	176/176	1.1	0.85–1.37	0.838
Synthetic textile dust	130/127	1.1	0.86–1.47	0.589
Flour dust	66/87	0.8	0.57–1.12	0.226
Leather dust	57/57	1.1	0.78–1.68	0.803
B-cell lymphoma				
Unexposed	1008/1436	1.0	-	-
Wood dust (any)	217/276	1.0	0.81–1.28	0.342
Hardwood dust	73/96	1.0	0.69–1.36	0.961
Softwood dust	121/144	1.1	0.81–1.40	0.410
Textile dust (any)	182/203	1.2	0.93–1.48	0.418
Natural textile dust	149/176	1.1	0.84–1.40	0.948
Synthetic textile dust	111/127	1.2	0.88–1.54	0.538
Flour dust	49/87	0.7	0.51–1.07	0.194
Leather dust	46/57	1.2	0.77–1.74	0.927
Diffuse large B-cell lymphoma				
Unexposed	319/1436	1.0	-	-
Wood dust (any)	56/276	1.1	0.77–1.56	0.294
Hardwood dust	19/96	1.0	0.57–1.67	0.719
Softwood dust	37/144	1.2	0.80–1.82	0.303
Textile dust (any)	62/203	1.4	1.00–1.98	0.097
Natural textile dust	45/176	1.1	0.76–1.93	0.700
Synthetic textile dust	33/127	1.2	0.77–1.82	0.377
Flour dust	12/87	0.6	0.33–1.14	0.109
Leather dust	7/57	0.7	0.31–1.59	0.205
Follicular lymphoma				
Unexposed	149/1436	1.0	-	-
Wood dust (any)	20/276	0.8	0.47–1.39	0.541
Hardwood dust	8/96	0.8	0.38–1.80	0.707
Softwood dust	13/144	0.8	0.44–1.56	0.494
Textile dust (any)	24/203	0.8	0.48–1.37	0.265
Natural textile dust	21/176	0.8	0.48–1.37	0.123
Synthetic textile dust	16/127	0.9	0.50–1.60	0.439
Flour dust	8/87	0.8	0.37–1.68	0.476
Leather dust	11/57	2.1	1.01–4.20	0.267
Chronic lymphocytic leukemia				
Unexposed	197/1436	1.0	-	-
Wood dust (any)	58/276	1.1	0.72–1.95	0.422
Hardwood dust	23/96	1.3	0.78–2.24	0.256
Softwood dust	31/144	1.2	0.74–1.85	0.304
Textile dust (any)	42/203	1.4	0.93–2.08	0.223
Natural textile dust	34/176	1.3	0.83–2.01	0.369
Synthetic textile dust	27/127	1.5	0.90–2.36	0.239
Flour dust	12/87	0.9	0.46–1.67	0.569
Leather dust	6/57	0.5	0.21–2.25	0.084
Multiple myeloma				
Unexposed	133/1436	1.0	-	-
Wood dust (any)	34/276	1.0	0.60–1.51	0.441
Hardwood dust	11/96	1.0	0.50–2.02	0.812
Softwood dust	17/144	1.0	0.57–1.81	0.551
Textile dust (any)	29/203	1.2	0.77–1.97	0.359
Natural textile dust	27/176	1.4	0.84–2.23	0.371
Synthetic textile dust	19/127	1.3	0.76–2.34	0.463
Flour dust	5/87	0.5	0.19–1.22	0.182
Leather dust	8/57	1.2	0.54–2.65	0.664
Hodgkin lymphoma				
Unexposed	225/1436	1.0	-	-
Wood dust (any)	24/276	0.7	0.43–1.19	0.278
Hardwood dust	10/96	0.6	0.31–1.35	0.287
Softwood dust	19/144	0.8	0.47–1.45	0.701
Textile dust (any)	21/203	1.2	0.71–2.10	0.221
Natural textile dust	18/176	1.2	0.68–2.17	0.423
Synthetic textile dust	12/127	1.1	0.55–2.11	0.813
Flour dust	13/87	1.2	0.60–2.32	0.983
Leather dust	7/57	1.3	0.52–3.18	0.609

With regard to the individual metrics of exposure to textile dust, DLBCL risk did not show an upward trend by intensity, frequency, or duration of exposure (supplementary material, www.sjweh.fi/show_abstract. php?abstract_id=3925, table S2d), nor did risk vary by type of textile dust, whether from natural or synthetic textiles (supplementary tables S2e–2f). There was no association between textile dust exposure and risk of lymphoma overall, B-cell lymphoma, nor any of the other major lymphoma subtypes we could analyze. FL risk did not increase by intensity (P=0.187), frequency (P=0.063), or duration of exposure (Wald test for trend: P=0.210) (supplementary table S2h) to leather dust. The Fisher method for combined probability testing resulted in a chance probability of an upward trend in FL risk with these three metrics of P=0.062.

Neither lymphoma (any subtype), nor B cell lymphoma, nor any of the other lymphoma subtypes showed increased risks suggestive of an association with exposure to leather dust or wood dust. Risks were all consistently inverse for ever exposure and cumulative exposure to flour dust, although for none it was possible to exclude chance as an explanation (supplementary tables S2a-c and 2g). Results did not change when replacing farm work with ever exposure to pesticides in the regression model (data not shown).

When excluding subjects ever exposed to solvents and subjects who had ever been working in a farm (table 3), risk of lymphoma (any subtype), and particularly B-cell lymphoma (accounting for 77% of all lymphoma in this analysis) was increased in association with ever exposure to leather dust (B-cell lymphoma: OR 2.2, 95% CI 1.00–4.78). However, we did not observe an upward trend with increasing level of cumulative exposure (P=0.096), nor by frequency, intensity or duration of exposure, although the Fisher test for combined probabilities was of borderline significance (P=0.050). B-cell lymphoma subtypes contributing to the association included FL (OR 2.8, 95% CI 0.73–10.9; P for trend=0.429), and multiple myeloma (OR 2.4, 95% CI 0.61–9.71; P for trend=0.079), both represented by a number of cases too small for a profitable more in depth investigation of the association. Also, risk of HL showed a two-fold increase in risk (OR 2.0, 95% CI 0.95–4.30) and an upward trend with cumulative exposure to textile dust (Wald test for trend: P=0.023). Results were similar for exposure to natural (OR 2.0, 95% CI 0.90–4.44, Wald test for trend: P=0.045) and to synthetic textile dust (OR 1.6, 95% CI 0.63–4.02, Wald test for trend: P=0.197). A sensitivity analysis conducted by excluding one study center at a time confirmed the excess risk ranging 1.8–3.0 (supplementary table S3). We did not detect heterogeneity in risk across study centers (Cochran’s Q=3.23, DF=5, P=0.665), nor across lymphoma subtypes (Cochran’s Q=6.01, DF=4, P=0.074). Risk of HL increased consistently by duration (P=0.092), intensity (P=0.047), and frequency (P=0.032) of exposure to textile dust (table 4). The Fisher method for combined probability testing provided some evidence that all null hypotheses associated with the individual tests of an upward trend in risk of HL could be reasonably rejected (P=0.0068) (table 4). Notably, such excess risk was observed among subjects aged <31 years, the median age of HL cases, but not among subjects aged ≥31. This finding was consistent over the whole study population (age ≤30, OR 3.2, 95% CI 0.62–16.1; age ≥31, OR 0.5, 95% CI 0.25–0.96), and in the subgroup of the unexposed to solvents and/or farm work (age ≤30, OR 4.3, 95% CI 0.56–32.9; age ≥31, OR 0.7, 95% CI 0.38–1.27) (data not shown).

**Table 3:** Risk for the major lymphoma subtypes associated with ever exposure and P-value associated with the Wald test for trend by cumulative exposure to specific organic dusts, after excluding subjects ever engaged in farm work or ever exposed to solvents. Regression models are adjusted for age (continuous), gender, study centre, education (categorized as ≤8, 9–12, ≥13), ever worked in a farm, ever exposed to solvents, and ever suffering from atopy. [OR=odds ratio; CI=confidence interval.]

Exposure	Cases/controls	OR	95% CI	P-value for trend
All lymphomas			
Unexposed	734/804	1.0	-	-
Wood dust (any)	40/44	0.9	0.57–1.44	0.730
Hardwood dust	10/12	0.8	0.32–1.79	0.737
Softwood dust	17/22	0.7	0.37–1.36	0.459
Textile dust (any)	101/95	1.1	0.81–1.54	0.516
Natural textile dust	84/82	1.0	0.74–1.48	0.743
Synthetic textile dust	55/56	1.0	0.68–1.52	0.474
Flour dust	38/48	0.8	0.51–1.26	0.202
Leather dust	19/12	1.8	0.85–3.75	0.192
B-cell lymphoma				
Unexposed	562/804	1.0	-	-
Wood dust (any)	33/44	0.9	0.57–1.44	0.884
Hardwood dust	8/12	0.8	0.32–1.79	0.771
Softwood dust	14/22	0.7	0.37–1.36	0.532
Textile dust (any)	83/95	1.1	0.81–1.54	0.790
Natural textile dust	70/82	1.0	0.74–1.48	0.876
Synthetic textile dust	45/56	1.0	0.68–1.60	0.491
Flour dust	38/48	0.7	0.42–1.16	0.144
Leather dust	16/12	2.2	1.00–4.78	0.096
Diffuse large B-cell lymphoma				
Unexposed	194/804	1.0	-	-
Wood dust (any)	10/44	0.9	0.45–1.99	0.993
Hardwood dust	2/12	0.6	0.14–2.87	0.672
Softwood dust	4/22	0.7	0.23–2.01	0.448
Textile dust (any)	30/95	1.3	0.83–2.18	0.351
Natural textile dust	23/82	1.1	0.67–1.95	0.894
Synthetic textile dust	11/56	0.8	0.41–1.66	0.732
Flour dust	9/48	0.6	0.35–1.54	0.252
Leather dust	2/12	0.8	0.18–3.79	0.870
Follicular lymphoma				
Unexposed	88/804	1.0	-	-
Wood dust (any)	3/44	0.8	0.23–2.71	0.526
Hardwood dust	0/12	-	-	-
Softwood dust	1/22	0.5	0.06–3.57	0.430
Textile dust (any)	9/95	0.6	0.27–2.28	0.126
Natural textile dust	8/82	0.6	0.25–2.27	0.391
Synthetic textile dust	6/56	0.7	0.27–1.65	0.902
Flour dust	3/48	0.5	0.15–1.70	0.313
Leather dust	3/12	2.8	0.73–10.9	0.429
Chronic lymphocytic leukemia				
Wood dust (any)	95/804	1.0	-	-
Hardwood dust	6/44	0.8	0.30–2.03	0.543
Softwood dust	1/12	0.6	0.07–4.94	0.493
Textile dust (any)	3/22	0.8	0.22–2.91	0.684
Natural textile dust	17/95	1.4	0.73–2.51	0.422
Synthetic textile dust	15/82	1.5	0.75–2.83	0.615
Flour dust	12/56	2.0	0.95–4.06	0.100
Leather dust	8/48	1.2	0.52–2.77	0.758
Wood dust (any)	0/12	-	-	-
Multiple myeloma				
Wood dust (any)	71/804	1.0	-	-
Hardwood dust	7/44	1.2	0.48–3.00	0.461
Softwood dust	1/12	0.8	0.10–6.91	0.864
Textile dust (any)	1/22	0.3	0.05–2.71	0.556
Natural textile dust	13/95	1.0	0.49–1.92	0.847
Synthetic textile dust	13/82	1.2	0.60–2.41	0.916
Flour dust	8/56	1.2	0.50–2.66	0.196
Leather dust	2/48	0.4	0.09–1.60	0.202
Wood dust (any)	3/12	2.4	0.61–9.46	0.079
Hodgkin lymphoma				
Wood dust (any)	133/804	1.0	-	-
Hardwood dust	4/44	0.8	0.25–2.68	0.536
Softwood dust	1/12	0.4	0.04–3.74	0.430
Textile dust (any)	1/22	0.3	0.03–2.26	0.266
Natural textile dust	13/95	2.0	0.95–4.30	0.023
Synthetic textile dust	11/82	2.0	0.90–4.44	0.049
Flour dust	7/56	1.6	0.63–4.02	0.197
Leather dust	10/48	1.4	0.62–3.33	0.810
Wood dust (any)	2/12	1.3	0.22–7.22	0.801

**Table 4:** Risk of Hodgkin lymphoma by exposure metrics of textile dust and risk of follicular lymphoma by exposure metrics to leather dust; tests for trend and Fisher test for combined probabilities, after excluding subjects exposed to solvents or who ever worked in a farm. [OR=odds ratio; CI=confidence interval.]

Exposure	B-cell lymphoma			Hodgkin lymphoma			Test for trend (P-value)
	
	Cases/controls	OR	95% CI	Cases/controls	OR	95% CI	
Textile dust							
Unexposed				133/804	1.0		
Ever exposed				13/95	2.0	0.95–4.30	
Cumulative exposure							
Medium–low				8/47	1.9	0.74–4.77	2.268 (0.023)
Medium–high				4/48	2.3	0.78–6.56	
Intensity							
Low				9/57	2.0	0.83–4.70	1.987 (0.047
Medium				4/28	2.9	0.80–10.5	
High				0/10			
Frequency							
Low				2/8	2.1	0.30–14.6	2.146 (0.032)
Medium				4/25	2.4	0.65–8.85	
High				7/62	1.9	0.73–4.76	
Duration (years)							
1–2				5/19	2.7	0.76–9.41	1.686 (0.092)
3–7				4/23	2.5	0.72–8.34	
8–20				2/27	1.4	0.28–6.66	
≥21				2/26	1.5	0.30–7.26	
Fisher test							17.77 (0.0068)
Degrees of freedom							6
Leather dust							
Unexposed	562/804	1.0					
Ever exposed	16/12	2.2	1.00–4.78				
Cumulative exposure							
Low	5/5	1.8	0.50–6.39				1.665 (0.096)
Medium–low	6/3	2.9	0.71–11.9				
Medium–high	4/2	2.8	0.50–15.2				
High	1/2	1.1	0.10–12.6				
Intensity							
Low	9/5	2.6	0.86–7.96				1.377 (0.169)
Medium	7/6	2.1	0.67–6.59		
High	0/1						
Frequency							
Low	4/1	6.0	0.65–54.7				1.221 (0.222)
Medium	10/6	2.9	1.02–8.38				
High	2/5	0.6	0.12–3.21				
Duration (years)							
1–2	5/6	1.4	0.40–4.65				1.973 (0.049)
3–7	2/1	3.0	0.26–35.1		
8–20	4/6	7.2	0.79–65.4				
≥21	5/5	2.0	0.51–7.62				
Fisher test							12.60 (0.050)
Degrees of freedom							6

Excluding from the analysis subjects ever exposed to solvents and subjects who had ever been working in a farm did not support the association between exposure to textile dust and risk of DLBCL. Results after the exclusions did not change in respect to the fully adjusted regression models for any of the lymphoma groups and subtypes we analyzed in relation to exposure to wood dust and flour dust (table 3)

Again, results did not change when excluding subjects ever exposed to pesticides instead of farm work (data not shown).

## Discussion

Our results provide tentative evidence of an increased risk of some lymphoma subtypes associated with exposure to specific organic dusts. There was an increase in risk of FL among subjects ever exposed to leather dust but no exposure-response trend. The association between exposure to textile dust and risk of DLBCL was only observed with hospital controls (supplementary table S1); it was similar, though weakened because of smaller numbers, after excluding subjects exposed to solvents and those who ever worked in a farm. In addition, it did not increase with any of exposure metrics either before (Fisher test: P=0.101) (supplementary table S2d) or after (data not shown) exclusions.

After excluding farm workers and subjects exposed to solvents from the analysis, results were inconclusive for an association between risk of B-cell lymphoma and exposure to leather dust, while the number of cases was too small to allow further analysis on FL risk and MM risk. Risk of HL emerged in relation to exposure to textile dust (OR 2.0, 95% CI 0.90–4.44), with consistently significant upward trends by the four exposure metrics we used in this study.

Several previous studies reported an excess risk of non-Hodgkin lymphoma among leather workers (27–29) and specifically FL (30); leather dust was among the suspected agents. However, a large multicenter study did not confirm the association (31). Our results support the only previous report of an association between risk of Hodgkin lymphoma and fabric dust in a large Canadian case–control study (32). On the other hand, our results are not consistent with previous publications (6, 9) suggesting that exposure to wood dust might contribute to increasing risk of some mature B-cell lymphoma subtypes, such as CLL and MM.

The International Agency for Research on Cancer (IARC) Monograph N. 100C confirmed the classification of wood dust and leather dust as group 1 human carcinogens, based mainly on the evidence for nasal cavity and nasopharyngeal cancer (33, 34). According to the IARC evaluation, the overall epidemiological findings would not provide evidence of an association of wood dust and leather dust with cancer of other sites. Exposures in the textile industry, including cotton, wool, flax and hemp dust, were considered in the IARC Monograph N. 48 (35), which concluded for limited evidence of an increase in risk of cancer of the nasal cavity among weavers (group 2B). Epidemiological findings were inconsistent for cancers other than the nasal cavity, including HL and NHL (35).

Enzymes, proteins and additives in flour dust can cause non-allergic and allergic reactions, such as baker’s asthma, among exposed workers (36). On the other hand, an excess risk of head and neck cancer was described among male, but not female, workers exposed to flour dust (37); also, exposure to flour dust was included among the high molecular weight allergens that were inversely related to risk of lymphoma (16, 17). We considered atopy as a possible confounder; however, in our multivariate analysis, having ever suffered from an atopic condition did not affect risk of lymphoma; and – for none of the inverse associations between exposure to flour dust and risk of lymphoma and its subtypes – was it possible to rule out chance as the determinant.

The organic dust definition includes a vast array of different agents, of diverse origin, with different physiochemical properties. Therefore, as we had a more precise definition of organic dust exposure, we refrained from using the generic category as the exposure variable. However, exposures in workplaces where organic dusts occur can be quite diverse: various types of dust may mix up with a range of different chemicals in wood preserving, in synthetic and natural textile fabricating from vegetal or animal fibers, and in leather curing and tanning. Besides, fungal and bacterial contaminants would develop in conditions of elevated moisture and temperature; and exposure to endotoxin, a component of the outer membrane of gram-negative bacteria, can also occur (38). Therefore, the question is whether the responsible agent would be a component of the organic dusts themselves, or other associated exposures. For instance, exposure to endotoxin originates a strong inflammatory response in atopic subjects accompanied by secretion of proinflammatory cytokines, including tumor necrosis factor alpha (TNF-α) (38). The hypothesis of a role of endotoxin exposure in the etiology of some lymphoma arises from the observation that TNF-α polymorphisms, resulting in TNF-α over-expression, are associated with an increase in DLBCL risk (39). However, household exposure to endotoxin did not show an association with NHL risk (40).

We adjusted our analysis for concurrent exposure to other known occupational causes of lymphoma, such as solvents, farm work, and pesticides, and we designed the unexposed reference by excluding subjects ever exposed to undefined organic dust, which would have allowed the effect of the specific organic dusts we focused on to emerge, if any existed. Also, we conducted analyses by excluding farm workers and subjects exposed to solvents, known to be at risk for lymphoma; and by excluding one study center at the time. Positive findings emerged after excluding from the analysis farm workers and subjects exposed to solvents; overlapping was marginal between both farm work and solvents on one side, and textile and leather dust on the other side, respectively, with the proportion of concurrent exposures ranging 6–14%, and it was highest for solvents and textiles, among DLBCL cases (17%) in respect to the controls (14%). We therefore consider that the apparent association between DLBCL risk and exposure to textile dust was due to bias from the imbalance in the distribution of co-exposure to solvents and textile dust by case–control status, not completely adjusted for in the multivariate regression analysis. On the other hand, the opposite might have happened in the distribution of co-exposure to solvents and textile dust between HL cases (8%) and the controls (14%), suggesting a bias in the opposite direction, again not completely adjusted for with the multivariate regression analysis. However, we cannot exclude chance as the explanation.

Limitations in interpreting our results include the small size of the study population in the analysis by lymphoma subtypes, which contributed to instability of the risk estimates associated with the categorical exposure metrics we used. Also, we made multiple comparisons; as a consequence, the few positive associations we observed might have arisen simply by chance. As expected, the response rate was lower among the population controls. We have no evidence of selection bias, as there was no significant difference between the two types of controls for the selected confounding variables. However, positive findings appeared mostly in the analyses with hospital controls (supplementary table S1).

Our study has also some strengths as the diagnosis was histologically verified for all the cases, and – instead of relying on self-reported exposures – expert industrial hygienists conducted the exposure assessment blinded to the case/control status of study subjects. The residual misclassification was likely to have been non-differential, and therefore likely to have impaired our ability to detect significant associations and exposure–response trends.

In conclusion, our analysis of a large European data set tentatively suggests that exposure to textile dust might contribute to increase risk of HL. Although difficult to interpret because of the small number of cases, our findings seem to suggest that subjects exposed to textile dust at younger age might be at higher risk of HL. Further research is warranted to clear up the interpretative doubts raised by the limitations in our study and to disentangle the effects of presumably protective conditions, such as atopy, and the possible carcinogenic action of specific organic dusts with due consideration of the confounding role of several other known occupational carcinogens in the etiology of lymphoma subtypes.

## Supplementary material

Supplementary material

## Protection of research participants

Following the Helsinki protocol for studies involving human subjects, all study subjects provided written consent before participation, in which they acknowledged that their identity could not be identified via the papers and that samples would have been fully anonymized.

Local Ethics Committees approved the study in each of the six study centers.

## References

[1] Ferlay J, Soerjomataram L, Ervik M, Eser S, Mathers C, Rebelo M Cancer Incidence and Mortality Worldwide;GLOBOCAN 2012 v1.0 (2013) Lyon:International Agency for Research on Cancer (IARC). IARC Cancer Base.

[2] Cocco P, t'Mannetje A, Fadda D, Melis M, Becker N, de Sanjosé S (2010). Occupational exposure to solvents and risk of lymphoma subtypes:results from the Epilymph case-control study. Occup Environ Med.

[3] Cocco P, Satta G, Dubois S, Pili C, Pilleri M, Zucca M (2013). Lymphoma risk and occupational exposure to pesticides:results of the Epilymph study. Occup Environ Med.

[4] Persson B, Fredriksson M, Olsen K, Boeryd B, Axelson O (1993). Some occupational exposures as risk factors for malignant lymphomas. Cancer.

[5] Fritschi L, Siemiatycki J (1996). Lymphoma, myeloma and occupation:results of a case-control study. Int J Cancer.

[6] Fritschi L, Benke G, Hughes AM, Kricker A, Vajdic CM, Grulich A (2005). Risk of non-Hodgkin lymphoma associated with occupational exposure to solvents metals, organic dusts and PCBs (Australia). Cancer Causes Control.

[7] Richardson DB, Terschüren C, Hoffmann W (2008). Occupational risk factors for non-Hodgkin's lymphoma:a population-based case-control study in Northern Germany. Am J Ind Med.

[8] Siemiatycki J, Richardson L, Gérin M, Goldberg M, Dewar R, Désy M (1986). Associations between several sites of cancer and nine organic dusts:results from an hypothesis-generating case-control study in Montreal, 1979-1983. Am J Epidemiol.

[9] Partanen T, Kauppinen T, Luukkonen R, Hakulinen T, Pukkala E (1993). Malignant lymphomas and leukemias and exposures in the wood industry:an industry-based case-referent study. Int Arch Occup Environ Health.

[10] Laakkonen A, Kyyrönen P, Kauppinen T, Pukkala EI (2006). Occupational exposure to eight organic dusts and respiratory cancer among Finns. Occup Environ Med.

[11] Matheson MC, Benke G, Raven J, Sim MR, Kromhout H, Vermeulen R (2005). Biological dust exposure in the workplace is a risk factor for chronic obstructive pulmonary disease. Thorax.

[12] Becker N, de Sanjose S, Nieters A, Maynadié M, Foretova L, Cocco PL (2007). Birth order, allergies and lymphoma risk:results of the European collaborative research project Epilymph. Leuk Res.

[13] Vajdic CM, Falster MO, de Sanjosè S, Martínez-Maza O, Becker N, Bracci PM (2009). Atopic disease and risk of non- Hodgkin lymphoma: an InterLymph pooled analysis. Cancer Res.

[14] Ellison-Loschmann L, Benavente Y, Douwes J, Buendia E, Font R, Alvaro T (2007). Immunoglobulin E levels and risk of lymphoma in a case-control study in Spain. Cancer Epidemiol Biomarkers Prev.

[15] Eriksson NE, Mikoczy Z, Hagmar L (2005). Cancer incidence in 13811 patients skin tested for allergy. J Investig Allergol Clin Immunol.

[16] Lindelöf B, Granath F, Tengvall-Linder M, Ekbom A (2005). Allergy and cancer. Allergy.

[17] Kennedy SM, Le Moual N, Choudat D, Kauffmann F (2000). Development of an asthma specific job exposure matrix and its application in the epidemiological study of genetics and environment in asthma (EGEA). Occup Environ Med.

[18] Mirabelli MC, Zock JP, D'Errico A, Kogevinas M, de Sanjosé S, Miligi L (2009). Occupational exposure to high molecular weight allergens and lymphoma risk among Italian adults. Cancer Epidemiol Biomarkers Prev.

[19] Espinosa A, Zock JP, Benavente Y, Boffetta P, Becker N, Brennan P (2013). Occupational exposure to immunologically active agents and risk for lymphoma:the European Epilymph case-control study. Cancer Epidemiol.

[20] Besson H, Brennan P, Becker N, Nieters A, De Sanjosé S, Font R (2006). Tobacco smoking alcohol drinking and non- Hodgkin's lymphoma:A European multicenter case-control study (Epilymph). Int J Cancer.

[21] Jaffe ES, Harris NL, Stein H, Vardiman JW, World Health Organization classification of tumours (2001). Pathology and genetics of tumours of hematopoietic and lymphoid tissues.

[22] Cocco P, t'Mannetje A, Fadda D, Melis M, Becker N, de Sanjosé S (2010). Occupational exposure to solvents and risk of lymphoma subtypes:results from the Epilymph case-control study. Occup Environ Med.

[23] 't Mannetje A, De Roos AJ, Boffetta P, Vermeulen R, Benke G, Fritschi L (2016). Occupation and Risk of Non-Hodgkin Lymphoma and Its Subtypes:A Pooled Analysis from the InterLymph Consortium. Environ Health Perspect.

[24] Cocco P, Satta G, Dubois S, Pili C, Pilleri M, Zucca M (2013). Lymphoma risk and occupational exposure to pesticides:results of the Epilymph study. Occup Environ Med.

[25] Kiran S, Cocco P, Mannetje A, Satta G, D'Andrea I, Becker N (2010). Occupational exposure to ethylene oxide and risk of lymphoma. Epidemiology.

[26] Fisher RA (1925). Statistical Methods for Research Workers.

[27] Siemiatycki J (2000). Risk Factors for Cancer in the Workplace Boca Raton.

[28] Decoufle P (1979). Cancer risks associated with employment in the leather and leather products industry. Arch Environ Health.

[29] Schenk M, Purdue MP, Colt JS, Hartge P, Blair A, Stewart P (2009). Occupation/industry and risk of non-Hodgkin's lymphoma in the United States. Occup Environ Med.

[30] Mester B, Nieters A, Deeg E, Elsner G, Becker N, Seidler A (2006). Occupation and malignant lymphoma:a population based case control study in Germany. Occup Environ Med.

[31] Neasham D, Sifi A, Nielsen KR, Overvad K, Raaschou-Nielsen O, Tjønneland A (2011). Occupation and risk of lymphoma:a multicentre prospective cohort study (EPIC). Occup Environ Med.

[32] Fritschi L, Siemiatycki J (1996). Lymphoma, myeloma and occupation:results of a case-control study. Int J Cancer.

[33] International Agency for Research on Cancer (IARC). Wood, leather and some associated industries (1981). IARC monographs on the evaluation of the carcinogenic risk to humans.

[34] International Agency for Research on Cancer (IARC) (2012). Radiation. Arsenic Metals, Fibres and Dusts. IARC monographs on the evaluation of the carcinogenic risk to humans.

[35] International Agency for Research on Cancer (IARC) (1990). Some Flame Retardants and Textile Chemicals and Exposures in the Textile Manufacturing Industry. IARC monographs on the evaluation of the carcinogenic risk to humans.

[36] Stobnicka A, Górny RL (2015). Exposure to flour dust in the occupational environment. Int J Occup Saf Ergon.

[37] Carton M, Menvielle G, Cyr D, Sanchez M, Pilorget C, Guizard AV, Icare Study Group (2018). Occupational exposure to flour dust and the risk of head and neck cancer. Am J Ind Med.

[38] Basinas I, Schlünssen V, Heederik D, Sigsgaard T, Smit LA, Samadi S (2012). Sensitisation to common allergens and respiratory symptoms in endotoxin exposed workers: a pooled analysis. Occup Environ Med.

[39] Rothman N, Skibola CF, Wang SS, Morgan G, Lan Q, Smith MT (2006). Genetic variation in *TNF* and *IL10* and risk of non-Hodgkin lymphoma: a report from the InterLymph Consortium. Lancet Oncol.

[40] Wang J, Cozen W, Thorne PS, Berhane K, Cerhan JR, Hartge P (2013). Household endotoxin levels and the risk of non-Hodgkin lymphoma. Cancer Causes Control.

