# Supplementary material for: Occupational exposure to organic dust and risk of lymphoma subtypes in the EPILYMPH case–control study
Source: Scand J Work Environ Health. 2020 Dec 16;47(1):42–51. doi: 10.5271/sjweh.3925 (PMC7801142; doi:10.5271/sjweh.3925)
Supplement: Supplementary material [file SJWEH-47-42-S001.pdf]

Occupational exposure to organic dust and risk of lymphoma subtypes in the EPILYMPH case–control study[1]

by Pierluigi Cocco, MD,[2] Giannina Satta, ScD, Federico Meloni, MD, Ilaria Pilia, MD,1 Fahad Ahmed, PhD, Nikolaus Becker, MD, Delphine Casabonne, PhD, Silvia de Sanjosé, PhD, Lenka Foretova, PhD, Marc Maynadié, MD, Alexandra -Nieters, PhD, Anthony Staines, MD, Andrea 't Mannetje, PhD, Mariagrazia Zucca, PhD, Maria Grazia Ennas, ScD, Marcello -Campagna, MD, Sara De Matteis, PhD, Yolanda Benavente, BS

1. *Supplementary material*
2. *Correspondence to: Prof. Pierluigi Cocco, Department of Medical Sciences and Public Health, Occupational Medicine unit, University of Cagliari, SS 554, km 4.500, 09042 Monserrato (Cagliari) Italy. [E-mail: pcocco@unica.it].*

Table S1. Risk for the major lymphoma subtypes associated with ever exposure and test for trend by cumulative exposure to organic dusts by type of controls.

Type of organic dust	*Any lymphoma*<br>*OR 95%CI*<br>*p for trend*	*B Cell lymphoma*<br>*OR 95%CI*<br>*p for trend*	*DLBCL*<br>*OR 95%CI*<br>*p for trend*	*FL*<br>*OR 95%CI*<br>*p for trend*	*CLL*<br>*OR 95%CI*<br>*p for trend*	*MM*<br>*OR 95%CI*<br>*p for trend*	*HL*<br>*OR 95%CI*<br>*p for trend*
Any wood dust (population controls)	0.8<br>0.59 – 1.14<br>*p*=0.512	0.8<br>0.58 – 1.17<br>*p*=0.571	0.9<br>0.51 – 1.57<br>*p*=0.993	0.6<br>0.28 – 1.26<br>*p*=0.212	1.0<br>0.55– 1.70<br>*p*= 0.969	1.1<br>0.55– 2.37<br>*p*= 0.251	0.8<br>0.38 – 1.70<br>*p*=0.823
Any wood dust (hospital controls)	1.1<br>0.87 – 1.51<br>*p*=0.109	1.2<br>0.89 – 1.62<br>*p*=0.096	1.3<br>0.82 – 2.11<br>*p*=0.040	1.3<br>0.57– 2.86<br>*p*=0.513	1.2<br>0.70– 2.04<br>*p*= 0.364	0.9<br>0.48– 1.58<br>*p*= 0.700	0.7<br>0.33 – 1.34<br>*p*=0.334
Any textile dust (population controls)	1.0<br>0.70 – 1.31<br>*p*=0.491	0.9<br>0.64 – 1.25<br>*p*=0.333	0.9<br>0.55 – 1.54<br>*p*=0.764	0.8<br>0.45– 1.54<br>*p*=0.221	1.3<br>0.73– 2.22<br>*p*=0.346	1.0<br>0.47 – 2.13<br>*p*=0.987	0.9<br>0.43 – 1.96<br>*p*=0.707
Any textile dust (hospital controls)	1.5<br>1.11 – 2.06<br>*p*=0.046	1.5<br>1.08 – 2.07<br>p=0.082	2.0<br>1.23 – 3.09<br>p=0.015	0.8<br>0.34 – 1.82<br>*p*=0.474	1.5<br>0.86 – 2.78<br>*p*=0.407	1.3<br>0.74 – 2.47<br>*p*=0.448	1.8<br>0.80 – 3.83<br>*p*=0.063

Flour dust (population controls)	0.7<br>0.43 – 1.11<br>*p*=0.078	0.6<br>0.37 – 1.06<br>*p*=0.065	0.6<br>0.22 – 1.29<br>*p*=0.117	0.8<br>0.29 – 1.98<br>*p*=0.384	1.0<br>0.46 – 2.32<br>*p*=0.706	*5 cases/ 87 controls*	1.1<br>0.43 – 2.88<br>*p*=0.661
Flour dust (hospital controls)	0.9<br>0.59 – 1.52<br>*p*=0.773	0.9<br>0.53 – 1.50<br>*p*=0.779	0.7<br>0.29 – 1.69<br>*p*=0.404	0.9<br>0.26 – 3.14<br>*p*=0.627	0.6<br>0.21 – 1.89<br>*p*=0.800	0.8<br>0.31– 1.23<br>*p*=0.920	1.3<br>0.48 – 3.34<br>*p*=0.786
Leather dust (population controls)	0.8<br>0.42 – 1.51<br>*p*=0.271	0.8<br>0.43– 1.63<br>*p*=0.338	0.2<br>0.03– 1.64<br>*p*=0.187	1.6<br>0.60 – 4.27<br>*p*=0.825	0.6<br>0.17 – 2.19<br>*p*=0.318	0.6<br>0.07 – 4.38<br>*p*=0.723	0.4<br>0.05– 3.33<br>*p*=0.315
Leather dust (hospital controls)	1.4<br>0.88 – 2.34<br>*p*=0.554	1.4<br>0.85– 2.44<br>*p*=0.438	1.1<br>0.46– 2.88<br>*p*=0.758	2.8<br>1.00 – 7.96<br>*p*=0.095	0.5<br>0.13 – 1.61<br>*p*=0.195	1.5<br>0.62 – 3.71<br>*p*=0.546	2.1<br>0.71– 5.96<br>*p*=0.660

Models adjusted for age, gender, study center, education (categorized as ≤8 years, 9-12 years, ≥ 13 years), ever worked in a farm, ever exposed to solvents, and ever suffering from atopy.

Tables S2. Risk of major lymphoma subtypes associated with ever exposure, cumulative exposure, duration, intensity, and frequency of exposure to six organic dusts. Covariates in the regression model include age (continuous), gender, study center, education, farm work, solvents, and atopy.

Table S2a. Wood dust (any).

Exposure metric	B-cell lymphoma<br>Cases/ctrls OR 95% CI	Diffuse Large B-cell Lymphoma<br>Cases/ctrls OR 95% CI	Follicular Lymphoma<br>Cases/ctrls OR 95% CI	Chronic Lymphocytic Leukaemia<br>Cases/ctrls OR 95% CI	Multiple myeloma<br>Cases/ctrls OR 95% CI	Hodgkin lymphoma<br>Cases/ctrls OR 95% CI
Unexposed	1008/1436 1.0 -	319/1436 1.0 -	149/1436 1.0 -	197/1436 1.0 -	133/1436 1.0 -	225/1436 1.0 -
Ever exposed	217/276 1.0 0.81-1.28	56/276 1.1 0.77-1.56	20/276 0.8 0.47-1.39	58/276 1.1 0.72-1.55	34/276 1.0 0.60-1.51	24/276 0.7 0.43-1.19
Cumulative exposure						
Low	47/82 0.77 0.52-1.13					
Medium-low	50/67 0.98 0.66-1.45	23/149 0.83 0.51-1.35	11/149 0.76 0.39-1.50	24/149 0.84 0.51-1.40	10/149 0.53 0.26-1.08	15/149 0.68 0.37-1.25
Medium-high	66/61 1.36 0.93-2.0	33/127 1.42 0.91-2.21	9/127 0.87 0.41-1.85	34/127 1.28 0.81-2.04	24/127 1.42 0.84-2.43	9/127 0.79 0.37-1.69
High	54/66 1.06 0.72-1.57					
Test for trend (p-	0.800 (0.424)	0.881 (0.378)	-0.938 (0.349)	0.754 (0.451)	1.059 (0.290)	-1.247 (0.212)
Intensity						
Low	105/142 0.91 0.68-1.22	21/142 0.77 0.47-1.27	12/142 0.84 0.44-1.63	31/142 1.07 0.67-1.72	15/142 0.79 0.42-1.47	10/142 0.44 0.25-1.06
Medium	84/100 1.20 0.86-1.66	25/100 1.49 0.90-2.46	6/100 0.84 0.34-2.04	21/100 1.12 0.64-1.96	13/100 0.99 0.51-1.92	11/100 1.03 0.50-2.14
High	28/34 1.01 0.60-1.71	10/34 1.47 0.70-3.10	2/34 0.60 0.14-2.60	6/34 0.84 0.33-2.10	6/34 1.57 0.61-4.03	3/34 0.85 0.23-3.06
Test for trend (p-	0.323 (0.747)	1.074 (0.283)	-1.150 (0.250)	-0,058 (0.954)	0.541 (0.589)	-0.958 (0.338)
Frequency						
Low	84/96 1.07 0.76-1.49	15/96 0.82 0.45-1.47	9/96 0.90 0.42-1.91	28/96 1.32 0.82-2.28	9/96 0.70 0.32-1.53	6/96 0.52 0.21-1.26
Medium	73/103 1.02 0.72-1.42	19/103 1.08 0.63-1.86	6/103 0.79 0.33-1.92	16/103 0.90 0.49-1.64	15/103 1.14 0.61-2.14	5/103 0.42 0.16-1.11
High	60/77 0.97 0.67-1.40	22/77 1.46 0.86-2.46	5/77 0.70 0.27-1.82	14/77 0.86 0.46-1.62	10/77 0.98 0.47-2.04	13/77 1.34 0.66-2.73
Test for trend (p-	-0.154 (0. 878)	0.899 (0.369)	-1.150 (0.250)	-0.489 (0.625)	0.423 (0.672)	-0.605 (0.545)
Duration						
1-2 years	58/89 0.93 0.65-1.33	14/89 0.85 0.47-1.55	6/89 0.74 0.31-1.77	12/89 0.81 0.42-1.57	7/89 0.68 0.30-1.55	8/89 0.52 0.24-1.16
3-7 years	44/64 0.87 0.58-1.31	12/64 1.01 0.52-1.94	5/64 0.85 0.32-2.24	14/64 1.02 0.53-1.95	4/64 0.44 0.15-1.28	9/64 1.11 0.49-2.49
8-20 years	56/67 1.11 0.76-1.63	16/67 1.27 0.71-2.28	3/67 0.53 0.16-1.75	15/67 1.20 0.65-2.23	9/67 1.15 0.54-2.46	4/67 0.54 0.18-1.59
21 years or more	59/56 1.22 0.82-1.82	14/56 1.39 0.74-2.62	6/56 1.21 0.49-3.00	17/56 1.24 0.67-2.29	14/56 1.63 0.84-3.17	3/56 1.09 0.32-3.72
Test for trend (p-	0.829 (0.407)	0.844 (0.399)	-0.673 (0.501)	0.815 (0.415)	1.221 (0.222)	-0.875 (0.382)

Table S2b. Hardwood dust

Exposure metric	B-cell lymphoma			Diffuse Large B-cell Lymphoma			Follicular Lymphoma			Chronic Lymphocytic Leukaemia			Multiple myeloma			Hodgkin lymphoma		
	Cases/ctrls	OR	95% CI	Cases/ctrls	OR	95% CI	Cases/ctrls	OR	95% CI	Cases/ctrls	OR	95% CI	Cases/ctrls	OR	95% CI	Cases/ctrls	OR	95% CI
Unexposed	1008/1436	1.0	-	319/1436	1.0	-	149/1436	1.0	-	197/1436	1.0	-	133/1436	1.0	-	225/1436	1.0	-
Ever exposed	73/96	1.0	0.69-1.36	19/96	1.0	0.57-1.67	8/96	0.8	0.38-1.80	23/96	1.3	0.78-2.24	11/96	1.0	0.50-2.02	10/96	0.6	0.31-1.35
Cumulative exposure																		
Low	14/28	0.74	0.38-1.43															
Medium-low	19/22	1.12	0.59-2.11	7/50	0.68	0.30-1.53	4/50	0.77	0.27-2.20	9/50	1.17	0.54-2.53	4/50	0.76	0.26-2.25	7/50	0.64	0.26-1.55
Medium-high	22/20	1.36	0.73-2.55	12/46	1.33	0.68-2.62	4/46	0.91	0.31-2.66	14/46	1.45	0.75-2.82	7/46	1.22	0.52-2.90	3/46	0.66	0.19-2.31
High	18/26	0.79	0.42-1.47															
Test for trend (p-	0.033 (0.974)			0.277 (0.782)			-0.353 (0.724)			1.056 (0.291)			0.231 (0.817)			-1.058 (0.290)		
Intensity																		
Low	19/30	0.83	0.46-1.50	3/30	0.46	0.14-1.54	2/30	0.58	0.14-2.52	6/30	1.33	0.53-3.36	3/30	1.03	0.30-3.54	2/30	0.28	0.06-1.26
Medium	29/42	0.94	0.57-1.54	8/42	1.00	0.45-2.20	4/42	1.11	0.38-3.28	11/42	1.48	0.71-3.07	2/42	0.40	0.09-1.71	6/42	0.92	0.35-2.42
High	25/24	1.20	0.67-2.15	8/24	1.67	0.72-3.85	2/24	0.76	0.17-3.35	6/24	1.10	0.43-2.85	6/24	1.99	0.75-5.28	2/24	0.91	0.19-4.32
Test for trend (p-	0.228 (0.820)			0.504 (0.614)			-0.310 (0.756)			0.803 (0.422)			0.429 (0.669)			-0.686 (0.493)		
Frequency																		
Low	12/24	0.66	0.33-1.35	1/24	0.19	0.03-1.44	1/24	0.34	0.05-2.58	6/24	1.61	0.63-4.12	2/24	0.81	0.18-3.58	0/24	-	-
Medium	19/25	1.03	0.55-1.91	5/25	1.07	0.40-2.86	4/25	1.78	0.58-5.42	5/25	1.27	0.46-3.51	2/25	0.77	0.17-3.43	3/25	0.52	0.14-1.94
High	42/47	1.10	0.71-1.72	13/47	1.37	0.71-2.65	3/47	0.67	0.20-2.26	12/47	1.22	0.61-2.47	7/47	1.19	0.50-2.84	7/47	1.17	0.47-2.93
Test for trend (p-	0.319 (0.750)			0.528 (0.597)			-0.337 (0.736)			0.725 (0.469)			0.214 (0.830)			-0.483 (0.629)		
Duration																		
1-2 years	15/27	0.82	0.42-1.58	3/27	0.58	0.17-1.94	2/27	0.86	0.20-3.75	2/27	0.50	0.11-2.21	2/27	0.76	0.17-3.41	4/27	0.54	0.17-1.70
3-7 years	24/19	1.57	0.84-2.92	5/19	1.25	0.46-3.43	4/19	1.84	0.60-5.71	10/19	3.02	1.31-6.93	2/19	0.97	0.21-4.35	4/19	1.14	0.33-3.94
8-20 years	17/26	0.85	0.45-1.60	5/26	0.94	0.35-2.50	1/26	0.40	0.05-3.05	6/26	1.29	0.51-3.31	4/26	1.44	0.48-4.38	0/26	-	-
21 years or more	17/24	0.79	0.42-1.51	6/24	1.24	0.49-3.13	1/24	0.37	0.05-2.85	5/24	0.90	0.33-2.48	3/24	0.85	0.24-2.97	2/24	1.44	0.31-6.61
Test for trend (p-	-0.295 (0.768)			0.181 (0.856)			-0.806 (0.420)			0.772 (0.440)			0.092 (0.926)			-0.812 (0.417)		

Table S2c. Softwood dust

Exposure metric	B-cell lymphoma<br>Cases/ctrls OR 95% CI	Diffuse Large B-cell Lymphoma<br>Cases/ctrls OR 95% CI	Follicular Lymphoma<br>Cases/ctrls OR 95% CI	Chronic Lymphocytic Leukaemia<br>Cases/ctrls OR 95% CI	Multiple myeloma<br>Cases/ctrls OR 95% CI	Hodgkin lymphoma<br>Cases/ctrls OR 95% CI
Unexposed	1008/1436 1.0 -	319/1436 1.0 -	149/1436 1.0 -	197/1436 1.0 -	133/1436 1.0 -	225/1436 1.0 -
Ever exposed	121/144 1.1 0.81-1.40	37/144 1.2 0.80-1.82	13/144 0.8 0.44-1.56	31/144 1.2 0.74-1.85	17/144 1.0 0.57-1.81	19/144 0.8 0.47-1.45
Cumulative exposure						
Low	27/49 0.73 0.45-1.20					
Medium-low	29/28 1.40 0.81-2.40	18/77 1.10 0.64-1.91	8/77 0.92 0.43-2.01	11/77 0.88 0.45-1.75	5/77 0.60 0.23-1.56	11/77 0.71 0.35-1.45
Medium-high	33/31 1.30 0.78-2.18	19/67 1.33 0.77-2.32	5/67 0.71 0.27-1.86	20/67 1.44 0.82-2.54	12/67 1.45 0.72-2.89	8/67 1.03 0.45-2.36
High	32/36 1.04 0.63-1.72					
Test for trend (p-	1.000 (0.317)	*1.001 (0.314)*	-0.467 (0.641)	1.014 (0.310)	0.632 (0.527)	-0.201 (0.841)
Intensity						
Low	53/74 0.92 0.63-1.34	16/74 1.00 0.57-1.78	6/74 0.70 0.29-1.68	12/30 0.96 0.49-1.85	6/74 0.75 0.31-1.79	9/74 0.67 0.31-1.44
Medium	41/44 1.23 0.78-1.93	12/44 1.35 0.68-2.65	5/44 1.24 0.47-3.32	13/44 1.59 0.80-3.17	5/44 0.91 0.34-2.45	8/44 1.22 0.52-2.90
High	27/26 1.20 0.69-2.11	9/26 1.58 0.71-3.51	2/26 0.66 0.15-2.89	6/26 1.06 0.41-2.72	6/26 1.97 0.75-5.19	2/26 0.66 0.14-3.08
Test for trend (p-	0.959 (0.338)	1.252 (0.210)	-0.203 (0.839)	0.780 (0.436)	0.762 (0.446)	-0.321 (0.748)
Frequency						
Low	41/52 1.00 0.65-1.55	13/52 1.17 0.62-2.23	5/52 0.80 0.30-2.09	10/52 1.08 0.52-2.24	3/52 0.55 0.16-1.82	5/52 0.62 0.23-1.68
Medium	35/42 1.03 0.66-1.70	10/42 1.13 0.55-2.31	4/42 0.91 0.31-2.65	9/42 1.24 0.57-2.70	7/42 1.41 0.60-3.32	4/42 0.50 0.16-1.53
High	45/50 1.12 0.73-1.72	14/50 1.32 0.70-2.48	4/50 0.80 0.28-2.33	12/50 1.20 0.60-2.41	7/50 1.11 0.47-2.63	10/50 1.41 0.64-3.13
Test for trend (p-	0.714 (0.475)	0.894 (0.372)	-0.327 (0.744)	0.650 (0.515)	0.543 (0.587)	0.087 (0.931)
Duration						
1-2 years	25/45 0.75 0.45-1.25	7/45 0.75 0.33-1.70	4/45 0.84 0.29-2.42	5/45 0.69 0.26-1.84	3/45 0.58 0.17-1.94	4/45 0.51 0.19-1.39
3-7 years	34/29 1.47 0.87-2.47	11/29 1.80 0.87-3.73	4/29 1.18 0.39-3.54	9/29 1.47 0.66-3.31	4/29 1.20 0.40-3.66	4/29 1.71 0.68-4.29
8-20 years	29/36 1.10 0.66-1.83	9/36 1.20 0.56-2.57	1/36 0.30 0.04-2.26	7/36 1.23 0.52-2.90	5/36 1.36 0.51-3.66	0/36 0.71 0.23-2.16
21 years or more	33/34 1.09 0.66-1.79	10/34 1.34 0.64-2.81	4/34 0.98 0.33-2.89	10/34 1.35 0.63-2.89	5/34 1.10 0.41-2.99	2/34 0.71 0.16-3.17
Test for trend (p-	0.920 (0.358)	1.149 (0.251)	-0.347 (0.728)	0.975 (0.330)	0.400 (0.689)	-0.246 (0.806)

Table S2d. **T**extile dust (any).

Exposure metric	B-cell lymphoma			Diffuse Large B-cell Lymphoma			Follicular Lymphoma			Chronic Lymphocytic Leukaemia			Multiple myeloma			Hodgkin lymphoma		
	Cases/ctrls	OR	95% CI	Cases/ctrls	OR	95% CI	Cases/ctrls	OR	95% CI	Cases/ctrls	OR	95% CI	Cases/ctrls	OR	95% CI	Cases/ctrls	OR	95% CI
Unexposed	1008/1436	1.0	-	319/1436	1.0	-	149/1436	1.0	-	197/1436	1.0	-	133/1436	1.0	-	225/1436	1.0	-
Ever exposed	182/203	1.17	0.93-1.48	62/203	1.41	1.00-1.98	24/203	0.84	0.51-1.37	42/203	1.39	0.93-2.08	29/203	1.24	0.77-1.97	21/203	1.22	0.71-2.10
Cumulative exposure																		
Low	55/53	1.37	0.92-2.04	17/53	1.43	0.79-2.57	10/53	1.44	0.70-2.98	17/53	2.21	1.21-4.01	5/53	0.87	0.33-2.26	8/53	1.38	0.58-3.24
Medium-low	46/50	1.25	0.82-1.90	16/50	1.44	0.80-2.61	8/50	1.11	0.50-2.47	6/50	0.97	0.40-2.36	11/50	2.14	1.05-4.37	4/50	0.79	0.27-2.36
Medium-high	39/47	1.04	0.67-1.63	15/47	1.50	0.82-2.77	3/47	0.43	0.13-1.42	12/47	1.53	0.77-3.04	5/47	0.88	0.33-2.30	5/47	1.60	0.58-4.45
High	42/53	1.02	0.66-1.55	14/53	1.28	0.69-2.39	3/53	0.39	0.12-1.29	7/53	0.80	0.35-1.85	8/53	1.15	0.52-2.56	4/53	1.26	0.42-3.78
Test for trend (p-	0.810 (0.418)			1.517 (0.129)			-1.303 (0.193)			0.630 (0.529)			1.173 (0.241)			1.010 (0.313)		
Intensity																		
Low	103/109	1.28	0.95-1.71	36/109	1.48	0.97-2.26	16/109	1.08	0.60-1.93	23/109	1.61	0.97-2.68	15/109	1.36	0.74-2.50	13/109	1.19	0.62-2.31
Medium	61/66	1.18	0.81-1.71	22/66	1.63	0.97-2.75	5/66	0.52	0.20-1.35	13/66	1.19	0.62-2.28	10/66	1.19	0.58-2.44	6/66	1.39	0.54-3.57
High	18/28	0.77	0.42-1.41	4/28	0.66	0.23-1.91	3/28	0.69	0.20-2.36	6/28	1.15	0.45-2.91	4/28	0.98	0.33-2.94	2/28	1.02	0.21-4.90
Test for trend (p-	0.791 (0.429)			1.177 (0.239)			-0.750 (0.453)			1.111 (0.267)			1.024 (0.306)			0.938 (0.348)		
Frequency																		
Low	27/30	1.19	0.70-2.05	8/30	1.09	0.55-2.54	5/30	1.19	0.44-3.18	*5/30*	*1.09*	*0.41-2.94*	4/30	1.32	0.45-3.91	5/30	1.21	0.43-3.49
Medium	43/39	1.51	0.96-2.38	17/39	2.16	1.18-3.94	6/39	1.26	0.51-3.11	*9/39*	*1.50*	*0.70-3.23*	5/39	1.05	0.40-2.77	6/39	2.16	0.80-5.81
High	112/134	1.06	0.80-1.41	37/134	1.27	0.84-1.92	13/134	0.65	0.34-1.22	*28/134*	*1.42*	*0.88-2.30*	20/134	1.28	0.74-2.22	10/134	0.97	0.47-2.01
Test for trend (p-	1.267 (0.205)			1.672 (0.095)			-0.625 (0.532)			1.720 (0.085)			1.284 (0.199)			0.791 (0.429)		
Duration																		
1-3 years	64/52	1.64	1.11-2.42	23/52	2.08	1.23-3.51	11/52	1.56	0.77-3.16	17/52	2.27	1.25-4.15	7/52	1.22	0.53-2.82	6/52	1.03	0.39-2.67
4-7 years	36/49	0.91	0.58-1.44	9/49	0.76	0.35-1.65	7/49	1.01	0.43-2.34	9/49	1.25	0.59-2.66	7/49	1.21	0.52-2.80	9/49	2.37	1.05-5.34
8-14 years	38/53	0.97	0.63-1.50	15/53	1.31	0.72-2.39	3/53	0.40	0.12-1.32	7/53	0.95	0.41-2.19	8/53	1.37	0.62-3.03	4/53	0.81	0.27-2.42
15 years or more	44/49	1.15	0.75-1.76	15/49	1.46	0.80-2.69	3/49	0.43	0.13-1.42	9/49	1.09	0.51-2.32	7/49	1.15	0.50-2.67	2/49	0.73	0.17-3.22
Test for trend (p-	0.714 (0.475)			1.230 (0.219)			-1.350 (0.177)			0.595 (0.552)			1.136 (0.256)			0.639 (0.523)		

Table S2e. Natural textile dust.

Exposure metric	B-cell lymphoma			Diffuse Large B-cell Lymphoma			Follicular Lymphoma			Chronic Lymphocytic Leukaemia			Multiple myeloma			Hodgkin lymphoma		
	Cases/ctrls	OR	95% CI	Cases/ctrls	OR	95% CI	Cases/ctrls	OR	95% CI	Cases/ctrls	OR	95% CI	Cases/ctrls	OR	95% CI	Cases/ctrls	OR	95% CI
Unexposed	1008/1436	1.0	-	319/1436	1.0	-	149/1436	1.0	-	197/1436	1.0	-	133/1436	1.0	-	225/1436	1.0	-
Ever exposed	149/176	1.09	0.84-1.40	45/176	1.11	0.76-1.63	21/176	0.81	0.48-1.37	34/176	1.29	0.83-2.01	27/176	1.37	0.84-2.23	18/176	1.22	0.68-2.17
Cumulative exposure																		
Low	36/45	1.26	0.79-2.01	11/45	1.34	0.62-2.92	6/45	2.41	0.72-8.02	9/45	1.60	0.61-4.21	5/45	0.90	0.38-2.11	8/45	1.12	0.32-3.87
Medium-low	46/42	1.32	0.70-2.49	13/42	1.36	0.49-3.77	10/42	2.37	0.55-10.2	8/42	2.20	0.66-7.28	9/42	0.93	0.27-3.22	2/42	1.83	0.42-7.97
Medium-high	36/40	1.83	0.98-3.40	13/40	1.82	0.68-4.85	2/40	3.81	0.97-15.1	12/40	2.32	0.68-7.87	6/40	2.04	0.68-6.14	5/40	0.54	0.08-3.60
High	31/49	1.40	0.74-2.65	8/49	1.88	0.71-5.02	3/49	0.77	0.12-4.85	5/49	2.98	0.25-9.39	7/49	1.12	0.34-3.68	3/49	2.35	0.49-11.3
Test for trend (p-	0.467 (0.641)			0.279 (0.780)			-0.970 (0.332)			0.753 (0.451)			1.458 (0.145)			0.793 (0.428)		
Intensity																		
Low	86/95	1.19	0.87-1.64	25/95	1.10	0.68-1.80	14/95	1.04	0.56-1.93	20/95	1.58	0.92-2.72	14/95	1.50	0.80-2.82	12/95	1.22	0.61-2.43
Medium	47/53	1.14	0.75-1.73	16/53	1.39	0.77-2.53	4/53	0.49	0.17-1.41	9/53	1.06	0.50-2.29	10/53	1.58	0.75-3.31	4/53	1.35	0.45-4.02
High	16/28	0.66	0.35-1.25	4/28	0.64	0.22-1.88	3/28	0.69	0.20-2.39	5/28	0.92	0.34-2.50	4/28	0.73	0.21-2.52	2/28	1.00	0.21-4.75
Test for trend (p-	0.296 (0.767)			0.243 (0.808)			-0.633 (0.527)			0.658 (0.510)			1.240 (0.215)			0.878 (0.380)		
Frequency																		
Low	22/27	1.08	0.60-1.95	5/27	0.66	0.23-1.91	3/27	0.81	0.24-2.76	5/27	1.22	0.45-3.30	3/27	1.08	0.31-3.69	5/27	1.51	0.51-4.44
Medium	28/31	1.25	0.74-2.13	9/31	1.37	0.64-2.96	6/31	1.42	0.57-3.58	4/31	0.89	0.30-2.63	5/31	1.58	0.59-4.27	3/31	1.06	0.29-3.88
High	99/118	1.04	0.77-1.40	31/118	1.16	0.74-1.81	12/118	0.66	0.34-1.28	25/118	1.42	0.85-2.36	19/118	1.38	0.78-2.43	10/118	1.16	0.55-2.42
Test for trend (p-	0.938 (0.349)			0.775 (0.439)			-0.390 (0.697)			1.370 (0.171)			1.700 (0.089)			0.914 (0.361)		
Duration																		
1-3 years	57/45	1.65	1.09-2.50	20/45	2.00	1.14-3.51	9/45	1.40	0.65-3.03	13/45	2.01	1.02-3.97	8/45	1.61	0.72-3.61	6/45	1.27	0.49-3.34
4-7 years	29/41	0.86	0.52-1.42	7/41	0.65	0.27-1.58	6/41	0.98	0.40-2.44	7/41	1.11	0.47-2.60	6/41	1.28	0.52-3.18	7/41	2.38	0.96-5.91
8-14 years	27/47	0.71	0.41-1.21	9/47	0.83	0.38-1.82	3/47	0.34	0.08-1.43	5/47	0.48	0.14-1.64	6/47	1.35	0.54-3.36	3/47	0.71	0.20-2.46
15 years or more	36/43	1.08	0.70-1.67	9/43	0.92	0.45-1.87	3/43	0.55	0.19-1.59	9/43	1.46	0.72-2.96	7/43	1.24	0.53-2.87	2/43	0.69	0.16-3.04
Test for trend (p-	0.089 (0.929)			-0.380 (0.704)			-1.020 (0.308)			0.701 (0.483)			1.150 (0.211)			0.474 (0.636)		

Table S2f. Synthetic textile dust.

Exposure metric	B-cell lymphoma			Diffuse Large B-cell Lymphoma			Follicular Lymphoma			Chronic Lymphocytic Leukaemia			Multiple myeloma			Hodgkin lymphoma		
	Cases/ctrls	OR	95% CI	Cases/ctrls	OR	95% CI	Cases/ctrls	OR	95% CI	Cases/ctrls	OR	95% CI	Cases/ctrls	OR	95% CI	Cases/ctrls	OR	95% CI
Unexposed	1008/1436	1.0	-	319/1436	1.0	-	149/1436	1.0	-	197/1436	1.0	-	133/1436	1.0	-	225/1436	1.0	-
Ever exposed	111/127	1.16	0.88-1.54	33/127	1.18	0.77-1.82	16/127	0.89	0.50-1.60	27/127	1.46	0.90-2.36	19/127	1.34	0.76-2.34	12/127	1.07	0.55-2.11
Cumulative exposure																		
Low	30/31	1.28	0.76-2.17	9/31	1.19	0.53-2.68	6/31	1.51	0.62-3.84	9/31	2.03	0.91-4.52	4/31	1.18	0.39-3.54	5/31	2.05	0.71-5.98
Medium-low	28/33	1.20	0.72-2.02	7/33	0.96	0.42-2.22	4/33	0.91	0.31-2.67	5/33	1.30	0.48-3.52	6/33	1.92	0.77-4.81	2/33	0.45	0.10-2.00
Medium-high	27/29	1.21	0.71-2.09	9/29	1.46	0.67-3.17	3/29	0.63	0.18-2.13	7/29	1.48	0.62-3.57	4/29	1.18	0.40-3.55	4/29	1.94	0.61-6.17
High	26/34	0.98	0.58-1.66	8/34	1.16	0.52-2.59	3/34	0.62	0.18-2.09	6/34	1.07	0.43-2.71	5/34	1.12	0.41-3.04	1/34	0.42	0.05-3.31
Test for trend (p-	0.882 (0.378)			0.675 (0.499)			-0.514 (0.608)			1.129 (0.259)			1.229 (0.219)			0.058 (0.934)		
Intensity																		
Low	71/79	1.21	0.86-1.71	21/79	1.15	0.68-1.95	11/79	1.04	0.53-2.07	17/79	1.57	0.87-2.81	12/79	1.48	0.75-2.90	9/79	1.50	0.55-2.59
Medium	36/40	1.19	0.74-1.90	11/40	1.43	0.70-2.89	4/49	0.64	0.22-1.88	8/40	1.24	0.54-2.84	7/40	1.36	0.57-3.26	3/40	0.99	0.28-3.52
High	4/8	0.61	0.18-2.04	1/8	0.51	0.06-4.14	1/8	0.79	0.10-6.52	2/8	1.50	0.30-7.36	0/8	-		0/8	-	
Test for trend (p-	0.912 (0.362)			0.522 (0.602)			-0.170 (0.865)			1.434 (0.151)			0.977 (0.329)			0.127 (0.899)		
Frequency																		
Low	23/35	1.10	0.51-2.37	8/35	1.06	0.33-3.38	2/35	1.13	0.19-6.82	3/35	0.97	0.23-4.14	3/35	2.67	0.71-9.99	3/35	2.24	0.38-13.4
Medium	46/51	1.63	0.82-3.23	13/51	1.49	0.51-4.33	11/51	4.24	1.12-16.1	12/51	2.92	2.95-9.01	12/51	0.84	0.17-4.05	5/51	2.39	0.43-13.3
High	42/41	1.95	0.92-4.16	12/41	1.64	0.51-5.28	3/41	1.74	0.32-9.35	12/41	4.65	1.31-16.5	12/41	3.44	0.83-14.4	4/41	1.90	0.27-13.4
Test for trend (p-	1.778 (0. 075)			0.916 (0.360)			0.113 (0.910)			2.510 (0.012)			1.607 (0.108)			0.287 (0.774)		
Duration																		
1-3 years	36/28	1.80	1.08-3.01	9/28	1.50	0.69-3.26	7/28	1.94	0.80-4.69	10/28	2.68	1.23-5.84	7/28	2.50	1.03-6.10	4/28	1.53	0.48-4.84
4-7 years	25/41	0.77	0.46-1.30	9/41	0.90	0.41-1.97	4/41	0.67	0.23-1.93	4/41	0.72	0.25-2.09	3/41	0.65	0.19-2.18	5/41	1.22	0.44-3.38
8-14 years	23/27	1.20	0.68-2.13	8/27	1.41	0.62-3.19	3/27	0.86	0.25-2.99	6/27	1.58	0.61-4.10	4/27	1.32	0.43-3.99	2/27	0.73	0.16-3.33
15 years or more	27/31	1.10	0.65-1.88	7/31	1.07	0.46-2.50	2/31	0.41	0.10-1.77	7/31	1.27	0.53-3.03	5/31	1.33	0.49-3.63	1/31	0.58	0.07-4.64
Test for trend (p-	0.712 (0.477)			0.430 (0.667)			-0.761 (0.447)			1.116 (0.264)			0.980 (0.327)			0.035 (0.972)		

Table S2g. Flour dust.

Exposure metric	B-cell lymphoma Cases/ctrls	OR 95% CI	Diffuse Large B-cell Lymphoma Cases/ctrls	OR 95% CI	Follicular Lymphoma Cases/ctrls	OR 95% CI	Chronic Lymphocytic Leukaemia Cases/ctrls	OR 95% CI	Multiple myeloma Cases/ctrls	OR 95% CI	Hodgkin lymphoma Cases/ctrls	OR 95% CI
Unexposed	1008/1436	1.0 -	319/1436	1.0 -	149/1436	1.0 -	197/1436	1.0 -	133/1436	1.0 -	225/1436	1.0 -
Ever exposed	49/87	0.74 0.51-1.07	12/87	0.61 0.33-1.14	8/87	0.79 0.37-1.68	12/87	0.87 0.46-1.67	5/87	0.48 0.19-1.22	13/87	1.18 0.60-2.32
Cumulative exposure												
Low	11/22	0.66 0.31-1.38										
Medium-low	11/23	0.69 0.33-1.44	7/45	0.72 0.32-1.62	5/45	0.90 0.34-2.37	3/45	0.45 0.13-1.50	2/45	0.37 0.09-1.58	11/45	1.62 0.75-3.50
Medium-high	13/20	0.83 0.41-1.69	5/42	0.51 0.20-1.31	3/42	0.65 0.20-2.16	9/42	1.27 0.59-2.73	3/42	0.60 0.18-2.00	2/42	0.50 0.11-2.26
High	14/22	0.78 0.39-1.55										
Test for trend (p-	-1.090 (0.276)		-1.695 (0.090)		-0.372 (0.710)		0.490 (0.624)		-1.174 (0.240)		-0.151 (0.879)	
Intensity												
Low	30/46	0.84 0.52-1.36	9/46	0.89 0.43-1.86	5/46	0.84 0.32-2.20	7/46	1.00 0.43-2.36	2/46	0.35 0.08-1.50	8/46	1.27 0.54-2.99
Medium	15/27	0.81 0.42-1.55	3/27	0.50 0.15-1.67	2/27	0.87 0.20-3.77	3/27	0.74 0.22-2.53	2/27	0.70 0.16-3.07	2/27	0.62 0.13-2.87
High	4/14	0.31 0.10-0.96	0/14	-	1/14	0.52 0.07-4.06	2/14	0.74 0.16-3.38	1/14	0.52 0.06-4.11	3/14	2.13 0.49-9.23
Test for trend (p-	-1.845 (0.065)		-1.986 (0.047)		-0.500 (0.617)		-0.519 (0.604)		-1.118 (0.264)		0.609 (0.542)	
Frequency												
Low	18/31	0.81 0.44-1.47	8/31	1.18 0.53-2.62	4/31	1.04 0.35-3.06	1/31	0.20 0.03-1.54	1/31	0.26 0.04-2.00	4/31	0.74 0.23-2.39
Medium	12/15	1.08 0.50-2.34	1/15	0.35 0.05-2.65	0/15	-	5/15	2.26 0.78-6.54	1/15	0.55 0.07-4.28	5/15	3.00 0.90-9.96
High	19/41	0.57 0.33-1.00	3/41	0.30 0.09-0.98	4/41	0.83 0.29-2.39	6/41	0.88 0.36-2.16	3/41	0.62 0.18-2.09	4/41	0.98 0.32-2.98
Test for trend (p-	-1.590 (0. 112)		-2.006 (0.45)		-0.434 (0.665)		-0.379 (0.705)		-1.046 (0.296)		0.634 (0.526)	
Duration												
1-2 years	13/26	0.85 0.33-1.29	3/26	0.51 0.15-1.71	3/26	0.85 0.25-2.92	1/26	0.23 0.03-1.73	2/26	0.58 0.13-2.54	*8/26*	*2.24 0.87-5.75*
3-5 years	8/26	0.56 0.26-1.18	2/26	0.36 0.09-1.55	2/26	0.73 0.17-3.14	2/26	0.53 0.12-2.32	2/26	0.77 0.18-3.33	*3/26*	*0.73 0.20-2.69*
5-11 years	10/16	0.44 0.17-1.10	3/16	0.78 0.23-2.72	0/16	-	2/16	0.85 0.19-3.82	1/16	0.51 0.07-3.98	*2/16*	*1.05 0.22-5.15*
12 years or more	18/19	1.53 0.78-3.02	4/19	0.93 0.31-2.77	3/19	1.48 0.42-5.17	7/19	2.12 0.85-5.28	0/19	-	*0/19*	-
Test for trend (p-	-0.597 (0.550)		-1.026 (0.305)		-0.209 (0.834)		-0.802 (0.423)		-1.505 (0.132)		-0.299 (0.765)	

Table S2h. Leather dust.

Exposure metric	B-cell lymphoma			Diffuse Large B-cell Lymphoma			Follicular Lymphoma			Chronic Lymphocytic Leukaemia			Multiple myeloma			Hodgkin lymphoma		
	Cases/ctrls	OR	95% CI	Cases/ctrls	OR	95% CI	Cases/ctrls	OR	95% CI	Cases/ctrls	OR	95% CI	Cases/ctrls	OR	95% CI	Cases/ctrls	OR	95% CI
Unexposed	1008/1436	1.0	-	319/1436	1.0	-	149/1436	1.0	-	197/1436	1.0	-	133/1436	1.0	-	225/1436	1.0	-
Ever exposed	46/57	1.16	0.77-1.74	7/57	0.71	0.31-1.59	11/57	2.06	1.01-4.20	6/57	0.51	0.21-1.25	8/57	1.20	0.54-2.65	7/57	1.29	0.52-3.18
Cumulative exposure																		
Low	12/14	1.21	0.55-2.67															
Medium-low	16/9	2.39	1.04-5.48	5/23	1.13	0.42-3.03	8/23	3.13	1.32-7.42	4/23	0.89	0.29-2.71	4/23	1.26	0.42-3.80	7/23	2.93	1.06-8.11
Medium-high	8/15	0.75	0.31-1.80	2/34	0.36	0.09-1.55	3/34	1.07	0.31-3.69	2/34	0.27	0.06-1.19	2/34	1.14	0.38-3.41	0/34	-	
High	10/19	0.88	0.36-1.77															
Test for trend (p-	-0.105 (0.916)			-1.283 (0.200)			1.113 (0.266)			-1.757 (0.079)			0.420 (0.674)			-0.514 (0.607)		
Intensity																		
Low	26/20	1.80	0.99-3.27	5/20	1.30	0.48-3.56	7/20	3.51	1.39-8.82	4/20	1.07	0.34-3.32	4/20	1.67	0.55-5.11	4/20	1.63	0.48-5.60
Medium	13/29	0.66	0.33-1.30	2/29	0.42	0.10-1.79	2/29	0.69	0.16-3.04	0/29	-		4/29	1.12	0.37-3.36	1/29	0.57	0.07-4.63
High	7/8	1.22	0.44-3.43	*0/8*	-		2/8	3.56	0.71-18.0	2/8	1.48	0.29-7.64	0/8	-		2/8	1.81	0.32-10.3
Test for trend (p-	-0.079 (0.937)			-1.449 (0.147)			1.320 (0.187)			-1.536 (0.125)			0.096 (0.924)			0.272 (0.786)		
Frequency																		
Low	7/9	1.13	0.41-3.07	1/9	0.79	0.09-5.81	1/9	1.39	0.17-11.7	1/9	0.58	0.07-4.78	2/9	1.85	0.39-8.91	3/9	3.37	0.74-15.5
Medium	18/20	1.50	0.77-2.90	3/20	1.02	0.29-3.54	4/20	3.22	1.01-10.3	1/20	0.23	0.03-1.80	4/20	1.75	0.56-5.49	1/20	0.49	0.06-4.23
High	21/28	0.96	0.53-1.72	3/28	0.53	0.16-1.79	6/28	1.74	0.68-4.46	4/28	0.70	0.23-2.09	2/28	0.61	0.14-2.64	3/28	1.15	0.31-4.31
Test for trend (p-	0.435 (0.663)			-1.006 (0.314)			1.857 (0.063)			-1.280 (0.200)			0.170 (0.865)			0.074 (0.941)		
Duration																		
1-2 years	15/19	1.04	0.52-2.09	2/19	0.56	0.13-2.44	5/19	2.31	0.82-6.50	1/19	0.28	0.04-2.16	3/19	1.17	0.33-4.11	2/19	1.28	0.26-6.40
3-5 years	10/8	1.61	0.62-4.16	2/8	1.36	0.28-6.65	1/8	1.32	0.15-11.4	4/8	1.89	0.54-6.68	1/8	0.95	0.11-8.01	1/8	1.44	0.13-16.3
5-11 years	9/14	1.00	0.43-2.36	1/14	0.41	0.05-3.19	4/14	3.07	0.95-9.93	1/14	0.45	0.06-3.60	1/14	0.73	0.09-5.81	4/14	2.06	0.58-7.30
12 years or more	12/16	1.18	0.55-2.56	2/16	0.82	0.18-3.68	1/16	0.89	0.11-7.06	0/16	-		3/16	1.74	0.48-6.35	0/16	-	
Test for trend (p-	0.460 (0.645)			-0.833 (0.405)			1.254 (0.210)			-1.667 (0.096)			0.576 (0.564)			0.110 (0.912)		

Table S3. Risk of Hodgkin lymphoma associated to ever exposure and to cumulative exposure to textile dust by study center, after excluding exposed to farm work and to solvents. Models are adjusted for age (continuous), gender, study centre, education (categorized as ≤8 years, 9-12 years, ≥ 13 years), ever worked in a farm, ever exposed to solvents, and ever suffering from atopy.

**Diagnosis**	**Unexposed Cas/ctrls**	**OR**	**Ever exposed Cas/ctrls**	**OR**	**95%CI**	**Test for trend with cumulative exposure (*p* value)**
**All Centers**	133/804	1.0	13/95	2.0	0.95-4.30	*0.023*
**Excluding Spain**	117/656	1.0	12/87	1.8	0.84-4.03	*0.080*
**Excluding France**	121/754	1.0	12/88	2.0	0.92-4.33	*0.025*
**Excluding Germany**	88/585	1.0	7/48	3.0	1.07–8.62	*0.018*
**Excluding Italy**	118/603	1.0	12/87	1.8	0.81–3.91	*0.062*
**Excluding Ireland**	115/730	1.0	12/88	2.2	0.98–4.85	*0.032*
**Excluding Czech republic**	106/692	1.0	10/77	1.8	0.79–4.26	*0.058*
